# Case Report: Buccal shifted flap with palatal “C” shape ridge split to facilitate the palatal bone augmentation without compromising the buccal vestibular depth: Report on three cases

**DOI:** 10.3389/fbioe.2025.1594325

**Published:** 2025-07-09

**Authors:** Mingfu Ye, Zhaozhao Chen, Wenjun Liu, Hom-Lay Wang

**Affiliations:** ^1^ Xiamen Key Laboratory of Oral Disease Diagnosis and Treatment, Affiliated Stomatological Hospital, Department of Implantological Center, Xiamen Medical College, Xiamen, China; ^2^ Shanghai Key Lab of D&A for metal-Functional Materials, School of Materials Science& Engineering, Tongji University, Shanghai, China; ^3^ Department of Periodontics and Oral Medicine, University of Michigan, Ann Arbor, MI, United States

**Keywords:** dental implant, palatal bone defect/deficiency, bone augmentation/reconstruction, flap management, ridge split, vestibular depth

## Abstract

Due to its dense connective tissue structure, the coronal advancement of the palatal flap is not feasible, making the reconstruction of single-site palatal bone defects particularly challenging. This case report describes the effectiveness and efficacy of an innovative technique combining the buccal shifted flap and palatal “C”-shaped ridge split together during bone augmentation procedures in the posterior maxilla. The described approach not only facilitates obtaining reliable bone regeneration without compromising the vestibular depth, but also surprisingly increases the horizontal contour.

## Introduction

There is an increasing number of patients seeking implant treatment to replace missing teeth, whether the loss is due to genetics, trauma, decay, or periodontal disease (Testori et al., 2024). One typical challenge in implant dentistry is the lack of sufficient bone to support a standard implant fixture. In addition, 1.5 mm–2 mm bone thickness around the dental implant is recommended for the long-term success (Monje et al., 2019). Numerous alveolar bone augmentation techniques have been introduced in the literature (P and asetti, 1994; Schliephake et al., 1991; Elboraey et al., 2025). Among these procedures, guided bone regeneration (GBR) is a well-established method for augmenting deficient alveolar ridges (Buser et al., 2009; Buser et al., 1990; Buser et al., 2000). Although widely utilized, it presents certain challenges (Wang and Boyapati, 2006), with primary closure being a critical factor.

In order to achieve primary closure, a standard approach is through the coronal advancement of the buccal/facial flap (Severi et al., 2000) (Smith, 2008). However, this is often associated with postoperative hematoma, swelling, and discomfort (Moslemi et al., 2016). More importantly, coronal advancement of the buccal flap will result in the coronal shift of the mucogingival junction and reduction of the vestibular depth (Aranda et al., 2015). This alteration may compromise the long-term health of dental implants by reducing the availability of at least 2 mm of keratinized mucosa (KM) and increasing muscle pull, both of which are crucial for peri-implant tissue stability. Consequently, additional soft tissue augmentation procedures may be required. The complexity increases in cases of maxillary palatal hard tissue deficiency, where flap management becomes more challenging and requires a more aggressive approach due to the limited elasticity of the palatal flap. Additionally, tension releasing for bounded single-site ridge augmentation is far more challenging than free-end multiple-site regions because the adjacent teeth impede the coronal advancement of the flap. The buccal shifted flap design has been reported to successfully reduce the morbidity of traditional flap management (Pohl et al., 2020; Tinti and Parma-Benfenati, 1995; Fugazzotto and De Paoli, 1999). Yet, to reconstruct the bounded palatal defect, traditional methods mainly focus on bone block transportation from the other donor sites (Yu et al., 2020) or titanium-reinforced PTFE membrane with biomaterials (Deeb et al., 2017; Deeb et al., 2016). Those approaches will inevitably counteract the beneficial effects of the above-mentioned flap management. Additionally, these techniques can be associated with significant postoperative morbidity. Thus, it is mandatory to figure out a solution that is manipulated locally and relative minimally invasive. A latest systemic review has concluded that the ridge-split technique (RST) is a reliable strategy to achieve horizontal bone (Vorovenci et al., 2024). While the previous literature is mainly focused on buccal bone augmentation, here we introduce the RTS on the palatal side.

Therefore, to minimize postoperative morbidity and reduce additional soft tissue augmentation procedures, we propose an innovative approach that combines the buccal sliding flap from the palatal site with the palatal “C” shape ridge split technique. This technique facilitates palatal bone augmentation while preserving vestibular depth.

## Materials and methods

Patients referred to our implant center with a palatal bone deficiency with normal gingival phenotype at maxillary posterior implant sites were included. The case report protocol was in accordance with CARE guidelines and the Declaration of Helsinki of 1965. Additionally, all participants provided informed consent. All of the surgeries were performed by one skilled oral surgeon at Xiamen Stomatological Hospital, affiliated to Xiamen Medical College.

Regarding the inclusion criteria, the following factors should be taken into consideration:

1. Adults ≥18 years of age
2. Non-smokers or <10 cigarettes/day
3. Systemically healthy or controlled systemic conditions: ASA I or II; diabetic patients may be included if HbA1c is well-controlled
4. Stable periodontal/implant condition: No active infection or inflammation at the surgical site.
5. Need for palatal bone augmentation: A single missing tooth, bounded by adjacent teeth, needs implant rehabilitation in the non-esthetic zone. CBCT demonstrates the severe palatal bone deficiency of the residual alveolar ridge while presenting a minimal buccal bone deficiency
6. Palatal soft tissue thickness >2.5 mm to ensure the blood supply
7. Good oral hygiene and good compliance

On the contrary, the exclusion criteria should consist of the following:

1. Patients <18 years of age
2. Heavy smokers: Often defined as >10 cigarettes/day
3. Uncontrolled systemic diseases: Uncontrolled diabetes, immunosuppression, bleeding disorder
4. Pregnant women
5. Uncontrolled topical or full-mouth periodontitis or other dental or neoplastic diseases
6. Poor oral hygiene or poor patient compliance
7. History of radiation therapy in the head/neck region

### Surgical protocol

**FIGURE 1:**
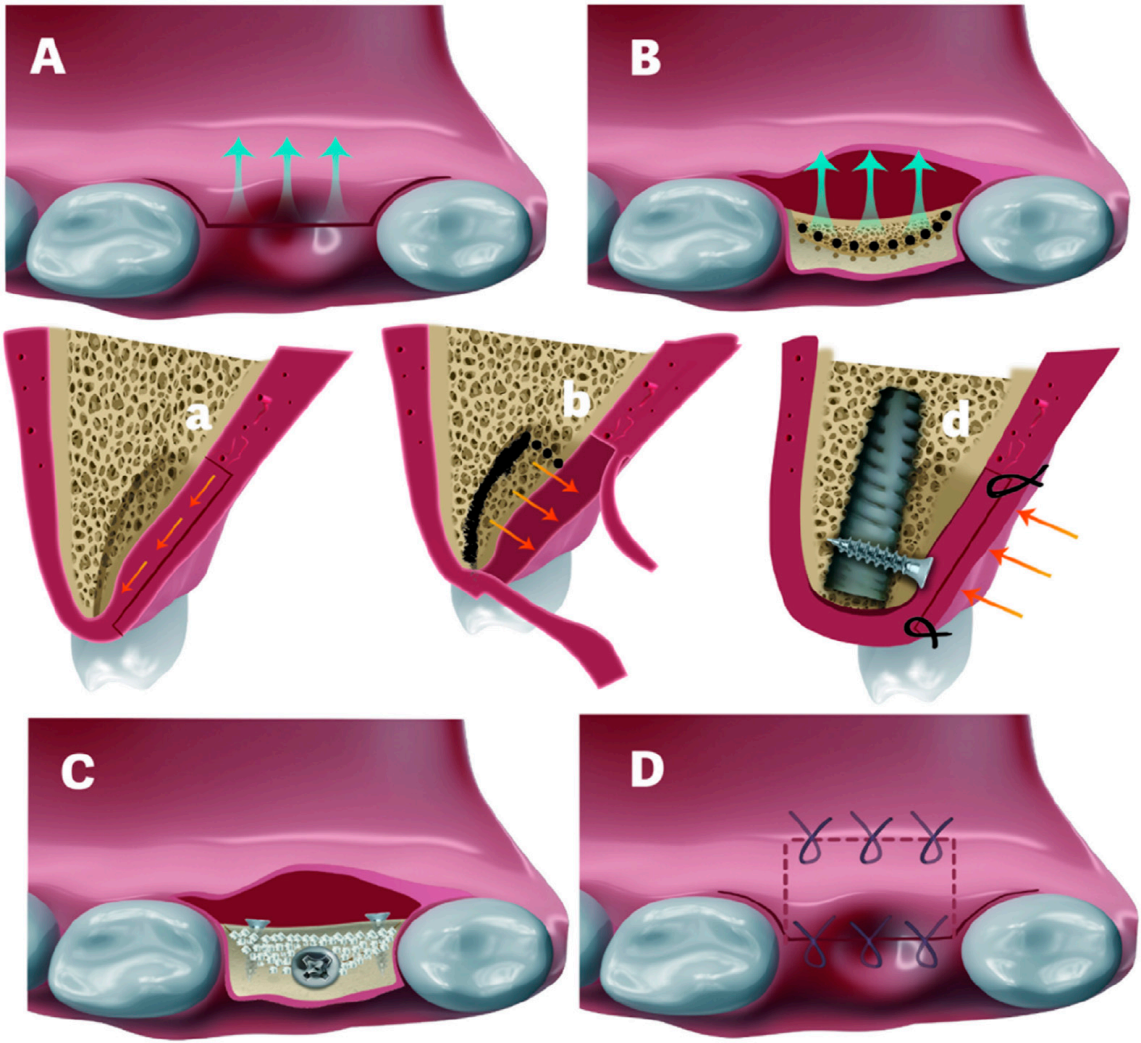
**(A)** and (a). Shifted flap preparation. A Z-shaped incision was used for the flap preparation in a. **(B)** and (b). C-shaped ridge split. Black-dotted lines refer to the penetration of the cortical bone to facilitate the ridge split. Black solid line in b indicates the direction of ridge split. **(C)**. Fixture installation and splitted bone block fixation with 2 titanium screws. White particles refer to bone graft in created space **(D)** and (d). Would closure. Palatal repositioned flap was secured by horizontal mattress sutures. Crestal incision was closed by interruped sutures in d.

The detailed flap design for a buccally shifted flap follows the approach described by Pohl et al. (2020) (Pohl et al., 2020). It begins with intrasulcular incisions on the palatal side of the adjacent teeth, connected by a depth of 1.5 mm linear palatal incision across the edentulous ridge. Flap preparation of subepithelial connective tissue is performed towards the median raphe of the palate, maintaining a minimum 1.5 mm thickness to prevent necrosis (Figure 1A a). Dissection extends 3–4 mm mesiodistally beyond the beyond the palatal aspect of the edentulous surgical site (Figure 1B), while the extent of median direction remains under 7–10 mm to ensure adequate blood supply, maintaining a 2:1 length-to-width ratio (Mormann and Ciancio, 1977). Two vertical incisions—one each at the mesial and distal aspects—are performed directly up to the bone at the palatal site, connected by a horizontal incision. Using a P24G periosteal elevator (Osung, South Korea), the connective tissue flap, including the periosteum, is reflected from mesial to distal until fully detached from the underlying bone (Figure 1b). The flap size is determined by the palatal vault anatomy to avoid injuring the greater palatine artery. In this approach, Vertical incisions and periosteal releasing at the buccal site are strictly avoided to preserve the blood supply. Instead, mild blunt releasing techniques (Hur et al., 2024; Abed et al., 2020)facilitate flap advancement for primary wound closure.

For palatal bone defect reconstruction, a palatally oriented “C”-shaped ridge split (Figure 1B b) was initiated by Precision Drill (TDRACT1, Nobel Biocare, United States), followed by using a larger luxation elevator (5#, Kangqiao Dental, Shanghai, China) tapping. To facilitate simultaneous implant placement, Implant site preparation was performed in a counter-clockwise direction, following an osseodensification manner rather than the conventional one. This approach not only enhances primary stability but also preserves the integrity of the palatal split bone. In certain cases, crestal sinus floor elevation (Ø 2.8/3.3 osteotome, concave and curved, Helmut Zepf, Germany) was conducted to further improve primary stability. This was typically achieved through primary apical bone engagement and subsequent fixation of the palatal split bone block. After implant placement, the migrated palatal bone block was secured with titanium screws (Figure 1C d), and the gap between the implant head and bone block was filled with bone graft (Bio-Oss, Geistlich, Switzerland) and covered with a collagen membrane (Bio-Gide, Geistlich, Switzerland). The palatal connective tissue was partially repositioned, ensuring at least 3 mm of width was covered by the remaining 1.5 mm-thick palatal epithelium to maintain pedicle blood supply. The incision was passively closed using two horizontal mattress sutures (Nylon 5-0, Jiahe) followed by two to three interrupted sutures (Figure 1D).

## Case reports

Here, the author presented 3 cases with buccally shifted flaps combined with palatal “C” shape ridge split and simultaneous implant placement.

### Case 1

**FIGURE 2:**
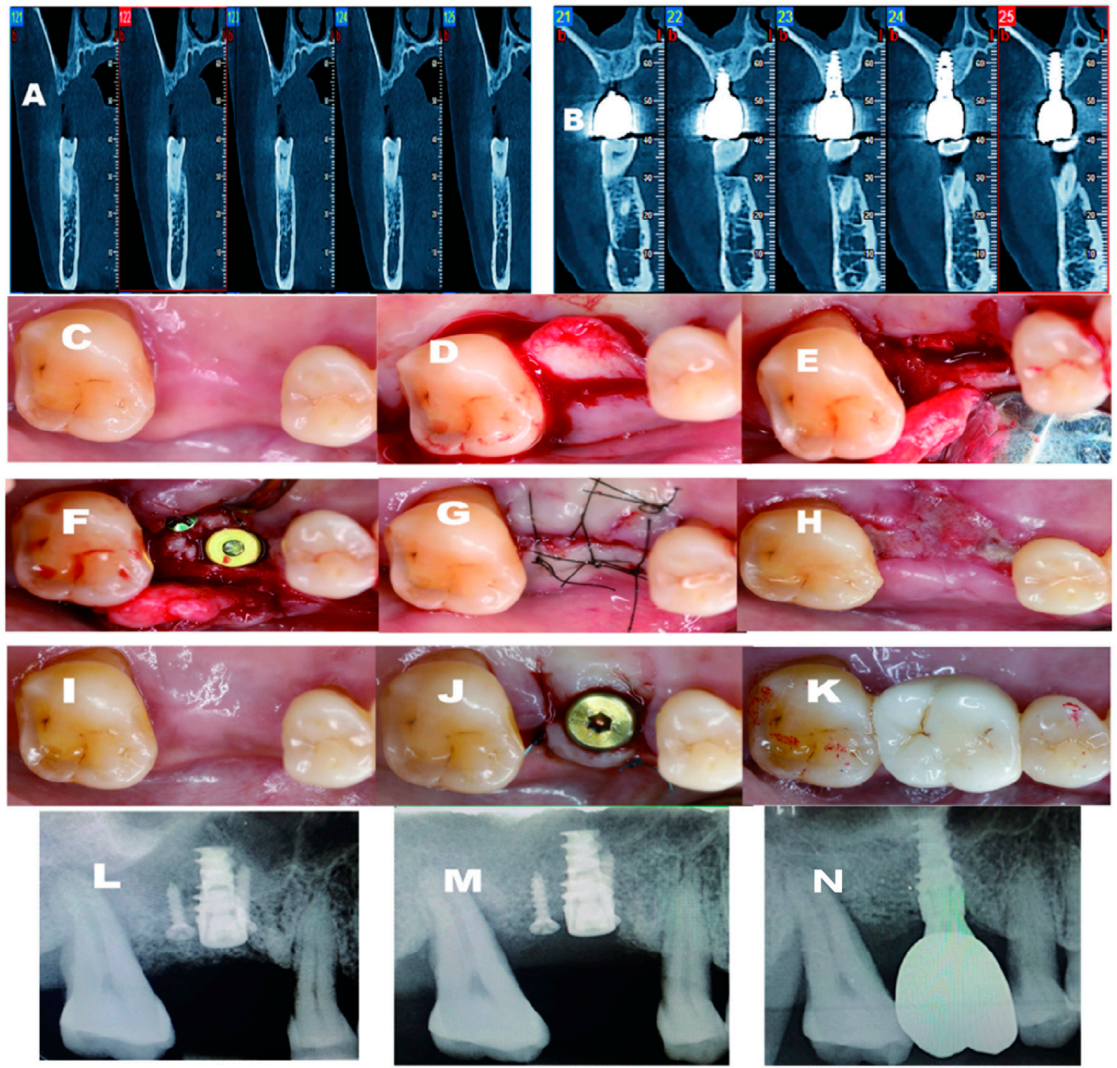
**(A)** Pre-operative CBCT indicates that large palatal defect; **(B)** 3 years follow-up CBCT. shows excellent bone regeneration; **(C)** Pre-operative-occlusal view; **(D)** Palatal flap elevated; **(E)** Ridge split initiaed; **(F)** Fixture inserted; **(G)** After sutured; **(H)** 2 weeks post-op; **(I)** 4-month post-opertative; **(J)** Second stage surgery and healing abutment installed; **(K)** Final crown delivered; **(L)** Periapical X-ray post-operative immediately; **(M)** Periapical X-ray 4 months after the surgery; **(N)** Periapical X-ray after crown delivery.

A 50-year-old woman presented with a missing tooth due to periodontitis for implant rehabilitation. After a thorough medical history evaluation and intraoral examination combined with a CBCT scan, A detailed treatment plan was presented to the patient, including a buccally sliding pedicle flap design, a palatal “C” shape ridge split, and simultaneous implant placement. A consent form was signed after the consultation. As mentioned earlier, after the partial-thickness flap was elevated (Figure 2). We can appreciate the palatal bone deficiency. Before starting the ridge splitting procedure, several holes were made to facilitate the splitting process, then connected with a saw disk with a diameter of 8 mm in order to separate the palatal bone medially. After the osteotomy and sinus floor transportation, a tapered implant from Nobel Biocare (Nebel Active, Nobel Biocare, United States) with a diameter of 4.3 mm*10 mm length was inserted, resulting in a primary stability of more than 25 N cm (torque wrench). A cover screw was installed immediately, followed by a titanium screw to secure the separated palatal bone. The gap and steps between the implant head and crest were grafted with Xenograft (Bio-oss, Geistlich, Switzerland) materials, covered by a collagen membrane (Bio-gide, Geistlich, Switzerland) using a regular GBR process. Healing was uneventful and the implant was restored after 6 months. In this case, no buccal vestibular depth change was detected. Surprisingly, buccal concavity was dramatically improved on the buccal aspect. From the CBCT scan, which was taken after 3 years of follow-up, we can appreciate that at least 2 mm of bone thickness was gained, resulting from the “C” shaped ridge split technique.

### Case 2

**FIGURE 3:**
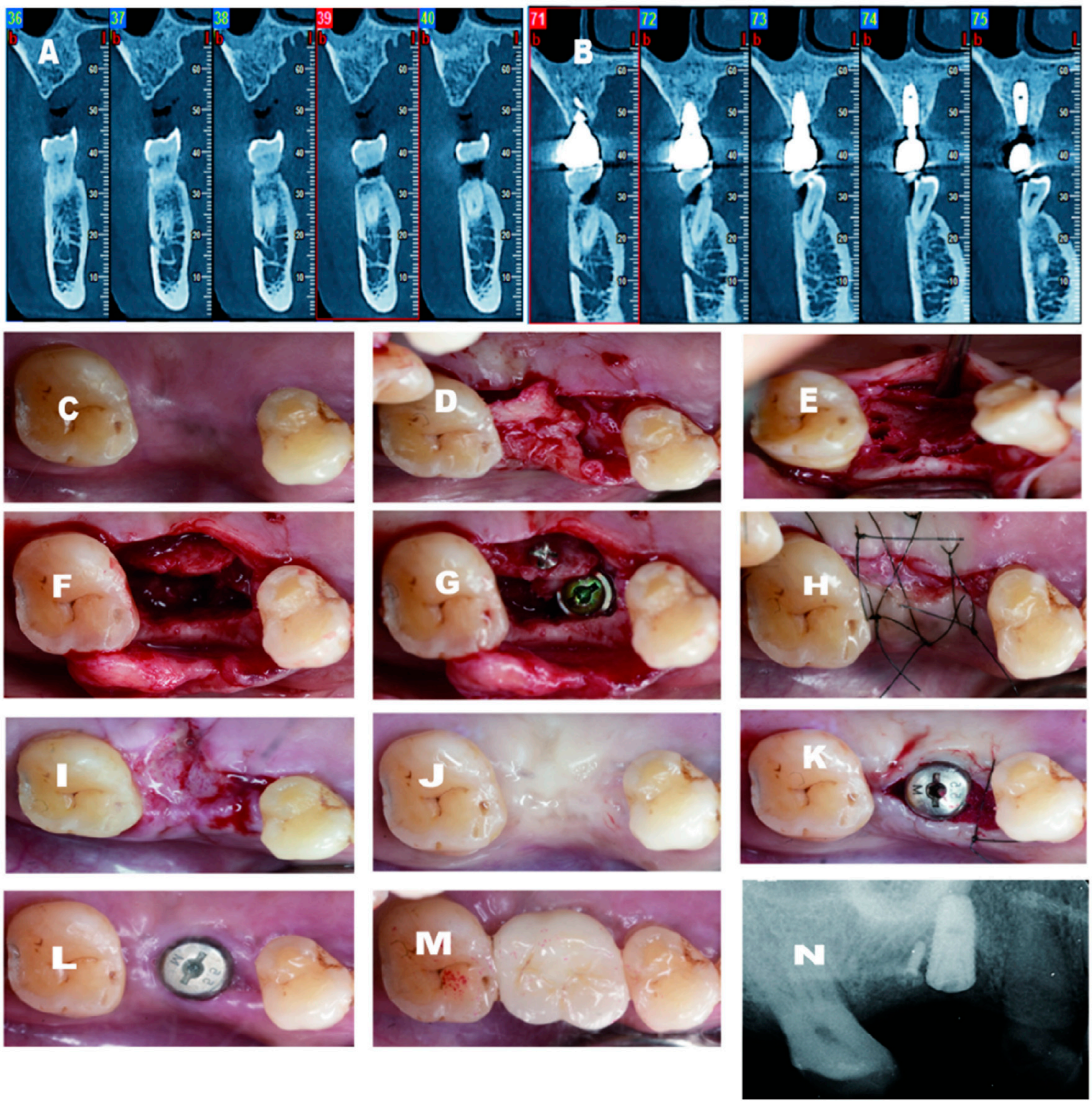
**(A)** Pre-operative CBCT demostrates large palatal defect; **(B)** 2 years follow-up CBCT shows excellent gain on the palatal aspect; **(C)** Pre-operative-occlusal view; **(D)** Palatal flap elevated; **(E)** C-shape scored; (F)Ridge split conducted; **(F)** Implant installed and titanium screw secured the migrated palatal bone; **(H)** Suture completed; (1)2 weeks after surgery; **(J)** 4 months after surgery; **(K)** Second stage surgery and healing abutment installed with secondary healing intension mesially; **(L,M)** 2 weeks after second stage and final crown delivery; **(N)** Periapical X-ray before second stage.

A healthy 62-year-old male was referred to my clinic with a missing upper right functional first molar (Figure 3). After a CBCT scan, a palatal bone defect was demonstrated. Similarly, the same technique was utilized, except that a different implant system was inserted. A 4.5*10 mm fixture from Dentium (Dentium Korea) was installed with good primary stability, with an insertion torque of more than 35N · cm (torque wrench). Two weeks after surgery, the implant site displayed good healing. The buccal vestibular depth was well-maintained. Additionally, 2–3 mm keratinized mucosa was gained on the buccal aspect. From the CBCT scan after 3 years of follow-up, the implant was maintained by at least 2 mm bone thickness.

### Case 3

**FIGURE 4:**
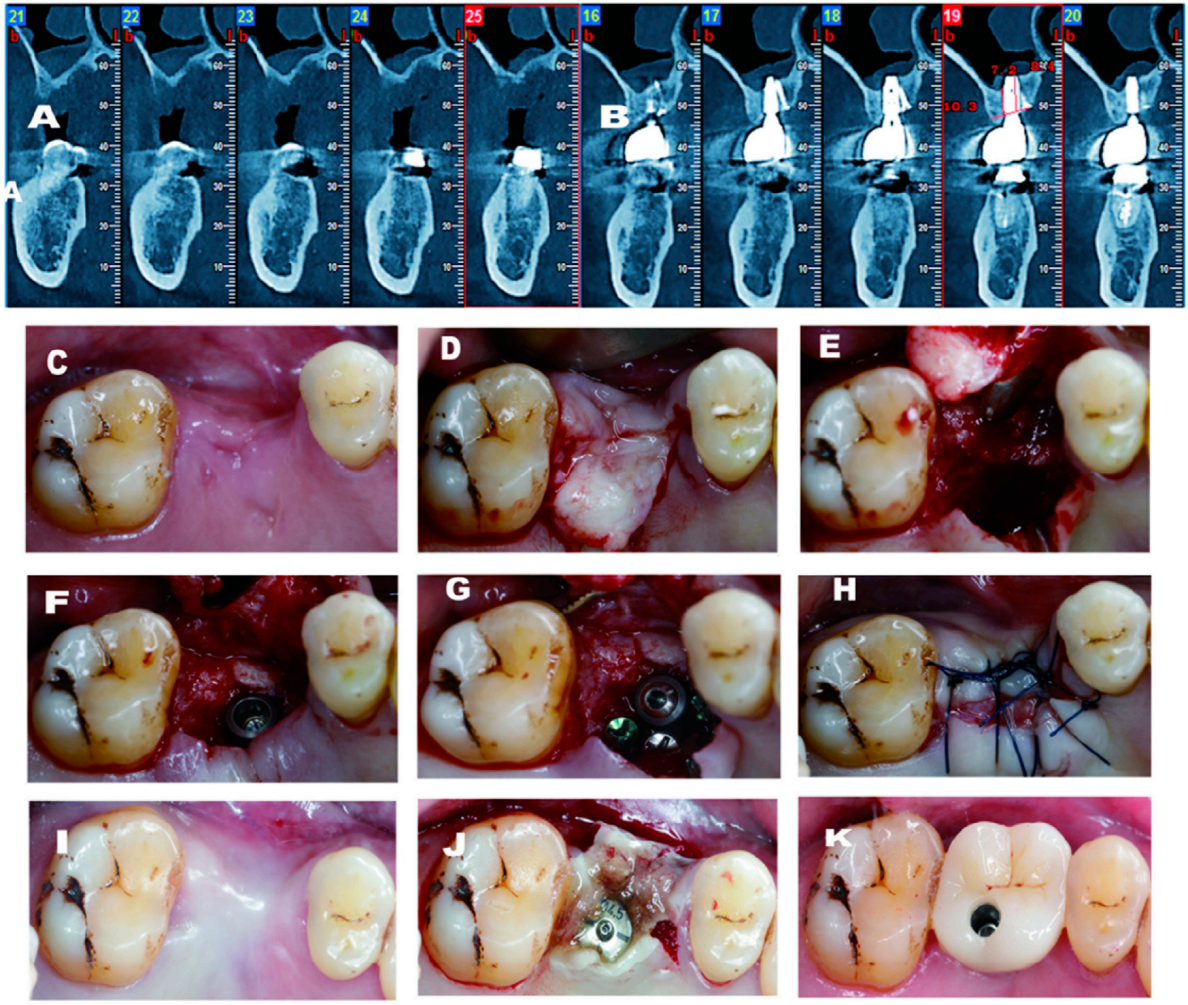
**(A)** Pre-operative CBCT demostrates large palatal and vertical defect; **(B)** 3 years follow-up CBCT shows excellent gain on both vertical and palatal aspects; **(C)** Pre-operative-occlusal view; **(D)** Palatal flap elevated; **(E)** Significant palatal defect after chipped palatal bone; **(F)** Implant installed after sinus floor transportation; **(H)** Non-primary closure after GBR; (1) 4 months after surgery; **(J)** Second stage surgery with tissue glew; **(K)** Final crown deliv- ery.

A 55-year-old man came to my center with a chief complaint of a missing tooth in the upper right maxilla. Minimal bone height and width were depicted in the CBCT scan. The same pre-operative protocol was administered to the patient (Figure 4). After the flap preparation, the residual bone width was limited. Even when trying to do ridge-splitting, the palatal bone chipped (Figure 4e). The implant was installed with a cover screw after crestal sinus floor elevation gain a good initial stability, with a insertion torque of more than 15N.cm (torque wrench). Three titanium screws were applied as tenting screw to augment the palatal defect. In this case, not only the buccal vestibular depth was well-maintained. But also, the severity of the buccal defect was alleviated from the benefit of the flap design. Regarding the hard tissue, the demand of 2 mm bone thickness around the palatal side was also achieved.

## Discussion

Many studies have shown that lateral bone augmentation using particulate xenograft and resorbable membrane can achieve predictable outcomes (Calciolari et al., 2000). However, in order to achieve predictable bone gain on the palatal sites, the rigidity and stability of the graft are crucial. This is particularly important due to challenges posed by the limited elasticity of surrounding tissues (Wysocki et al., 2011). While autogenous bone blocks remain the gold standard for significant bony defect reconstruction, they have notable drawbacks, including limited availability and donor-site morbidity (Liou et al., 2000).

In order to overcome these drawbacks, researchers have been continuously modifying and updating new approaches. The latest systemic review (Vorovenci et al., 2024) shows that the ridge-split technique can predictably gain an average horizontal bone gain of 3.6 mm with a relatively better outcome compared with osseodensification (Vorovenci et al., 2024). In addition, the ridge-splitting strategy facilitates dental implant insertion and space maintenance. More importantly, the exposed medullary bone in the split provides similarly autogenous conditions for new bone formation (Flanagan, 2024). The benefits of ridge split alone are apparent, but the crestal bone stability after this approach should not be underestimated (Palkovics et al., 2023). Several recent studies indicated that ridge split combined with guided bone regeneration (GBR) could dramatically reduce bone remodeling (Zhang and Huang, 2021; Issa et al., 2024; Bergamini et al., 2023). In these particular cases, we combined ridge split technique with GBR and finally achieved an excellent horizontal bone gain to engage the palatal aspects of the implants. With regard to the “C” shape toward palatal orientation rather than the straight ridge split, we took two essential factors into consideration. The first motivation is to protect the integrity of bone around adjacent teeth. Otherwise, it may induce undesired complications, including pulp irritation. The second intention is to facilitate the simultaneous implant placement without aggressively migrating the palatal bone block, which might result in graft necrosis (Wu et al., 2019). All the post-op CBCTs show the successful outcomes from ridge split and bone reconstruction. Postoperative pain was reported as mild to moderate and generally subsided within 7 days. All three patients expressed satisfaction with the outcome of the restoration. In these three cases, no fixation screw exposure was observed. This outcome may be attributed to the following three factors. First, the small sample size of the study may not be sufficient to detect potential complications. Second, the repositioning of the shifted connective tissue and collagen membrane likely preserved adequate blood supply and overall soft tissue thickness, thereby reducing the risk of screw exposure. Third, appropriate recipient site selection plays a critical role in minimizing complications. Although fixation screw exposure has been reported in the literature as a challenge in such procedures (Al Haydar et al., 2023), when it does occur, management strategies include removing the exposed device, debriding the wound, and covering it with a collagen sponge or membrane. In some cases, clinicians have opted to leave the exposed screw in place, for a minimal of 6–8 weeks, if there are no signs of infection.

In addition to hard tissue augmentation, the techniques employed in all three cases not only increased hard tissue volume but also enhanced soft tissue volume, eliminating the need for additional soft tissue surgeries. The buccal contour gains can mainly be attributed to the combined effects of the buccally shifted palatal connective tissue and the secondary healing of the buccal flap. The application of this flap design was able to create space for palatal defect augmentation and facilitate flap manipulation with less tension (Pohl et al., 2020; Tinti and Parma-Benfenati, 1995; Fugazzotto and De Paoli, 1999), ultimately precluding the need for secondary soft tissue management with surprisingly additional horizontal keratinized mucosa gain.

Limitations of the current study include, but are not limited to, uncalibrated photography techniques, a small sample size, potential patient selection bias, and the absence of quantitative measurements, all of which may limit the generalizability of the findings. Although all procedures were performed by an experienced clinician, the ridge-split technique was not fully standardized across the three cases, which could have influenced the final outcomes. Additionally, the lack of prosthetic-driven surgical guides may have contributed to improper alignment of the screw channels. Finally, ridge splitting with simultaneous implant placement carries increased risks of complications—such as fixation screw or implant exposure and subsequent infections (Al Haydar et al., 2023)—which require careful consideration by skilled surgeons.

## Conclusion

The developed regimen, combining ridge split and buccal-shifted flap with simultaneous implant placement, is a practical surgical approach to gain predictable outcome without compromising vestibular depth. Surprisingly, it beneficially alleviates the buccal soft tissue deficiency without additional surgery. In the future, we need further studies to justify this technique.

## Data Availability

The original contributions presented in the study are included in the article/supplementary material, further inquiries can be directed to the corresponding author.
